# A Novel Pathogenic Avipoxvirus Infecting Vulnerable Cook’s Petrel (*Pterodroma cookii*) in Australia Demonstrates a High Genomic and Evolutionary Proximity with South African Avipoxviruses

**DOI:** 10.1128/spectrum.04610-22

**Published:** 2023-02-07

**Authors:** Subir Sarker, Shane R. Raidal

**Affiliations:** a Department of Microbiology, Anatomy, Physiology and Pharmacology, School of Agriculture, Biomedicine and Environment, La Trobe University, Melbourne, Victoria, Australia; b School of Agricultural, Environmental and Veterinary Sciences, Faculty of Science and Health, Charles Sturt University, Wagga, New South Wales, Australia; University of Prince Edward Island

**Keywords:** *Avipoxvirus*, petrel, next-generation sequencing, transmission electron microscopy, phylogenetics, virus evolution

## Abstract

Avipoxviruses are assumed to be restricted to avian hosts and are considered to be important viral pathogens that may impact the conservation of many vulnerable or endangered birds. Recent reports of avipoxvirus-like viruses from reptiles suggest that cross-species transmission may be possible within birds and other species. Most of the avipoxviruses in wild and sea birds remain uncharacterized, and their genetic variability is unclear. Here, cutaneous pox lesions were used to recover a novel, full-length Cook’s petrelpox virus (CPPV) genome from a vulnerable Cook’s petrel (*Pterodroma cookii*), and this was followed by the detection of immature virions using transmission electron microscopy (TEM). The CPPV genome was 314,065 bp in length and contained 357 predicted open-reading frames (ORFs). While 323 of the ORFs of the CPPV genome had the greatest similarity with the gene products of other avipoxviruses, a further 34 ORFs were novel. Subsequent phylogenetic analyses showed that the CPPV was most closely related to other avipoxviruses that were isolated mostly from South African bird species and demonstrated the highest sequence similarity with a recently isolated flamingopox virus (88.9%) in South Africa. Considering the sequence similarity observed between CPPV and other avipoxviruses, TEM evidence of poxvirus particles, and phylogenetic position, this study concluded that CPPV is a distinct candidate of avipoxviruses.

**IMPORTANCE** Emerging viral disease is a significant concern with potential consequences for human, animal, and environmental health. Over the past several decades, multiple novel viruses have been found in wildlife species, including birds, and they can pose a threat to vulnerable and endangered species. Cook’s petrel is currently listed as vulnerable. The threats to the species vary, but are, to a large degree, due to anthropogenic impacts, such as climate change, habitat loss, pollution, and other disturbances by humans. Knowledge of viral pathogens, including poxvirus of Cook’s petrel is currently virtually nonexistent.

## INTRODUCTION

Over the past decades, seabird populations have declined faster than those of any other bird taxa (1, 2). Cook’s petrel (Pterodroma cookii) is one of a group of 10 small, pelagic petrel taxa. They breed in New Zealand on the Little Barrier and Great Barrier Islands, off the northeastern coast of New Zealand’s North Island, as well as Codfish Island, near Stewart Island (3); however, Cook’s petrels formerly bred throughout both the North and South Islands, on mountain tops and ranges (3). After hundreds of years of predation by introduced mammals, they became confined to just three islands. In the north, the largest breeding colony is on Little Barrier Island, with lesser numbers breeding on the nearby Great Barrier Island. In southern New Zealand, Cook’s petrels breed on Codfish Island, off the coast of Stewart Island (4). Cook’s petrel is currently listed as “vulnerable” under the International Union for Conservation of Nature’s (IUCN) criteria (5). The ongoing major threats to Cook’s petrel are introduced predators, which could be confounding drivers of this declining species. Additionally, there are ship rats, feral cats, and feral pigs on Great Barrier Island (6). Infectious diseases, including those caused by avipoxviruses, have also been identified as an important risk factor in the conservation of small and endangered populations, particularly in island species (7–11). The impact of the introduction of avipoxviruses has been severe for the avifauna of various archipelagos (12). For instance, the emergence of an avipoxvirus with a high prevalence (88%) in Hawaiian Laysan albatrosses (Phoebastria immutabilis) enabled one of the first detailed studies of the epidemiology and population-level impact of the disease in these seabirds (12, 13).

Avipoxviruses are large, double-stranded DNA (dsDNA) viruses that belong to the genus *Avipoxvirus* (family Poxviridae, subfamily Chordopoxvirinae) and may cause proliferative, diphtheritic, or systemic lesions in birds (14, 15). Although poxviruses have evolved to infect a wide range of host species, only 12 species have been approved under the genus *Avipoxvirus* to date (16). A further two viruses, namely, crowpox virus and peacockpox virus, are putative members of the genus *Avipoxvirus*, but they have not yet been approved as species by the International Committee on Taxonomy of Viruses (ICTV) (16). There are also several complete avipoxvirus genomes: two shearwaterpox viruses (SWPV1 and SWPV2) (11), two magpiepox viruses (MPPV and MPPV2) (17, 18), a mudlarkpox virus (MLPV) (19), penguinpox virus 2 (PEPV2) (10), two albatrosspox virus (ALPV and ALPV2) (8, 9), and a poxvirus in-house finches (Haemorhous mexicanus) (20) available in GenBank that are not yet ICTV recognized species. In addition, avipoxvirus infection has been found in at least 374 avian species and 23 orders of wild and domestic bird species (21–23), with many more avian hosts being considered susceptible. In general, avipoxviruses appear to have been present in bird populations for extended periods, leading to low levels of infection and relatively mild disease.

Relatively little is known about the general prevalence or effects of poxviruses in seabird species, including for Cook’s petrel (Pterodroma cookii). Therefore, the aim of the present study was to identify and characterize a novel poxvirus during clinical investigation on samples collected from cutaneous lesions of a vulnerable Cook’s petrel that was found in Southern Queensland, Australia, in 2022.

## RESULTS

### Pathological evidence of avipoxvirus in Cook’s petrel.

Cutaneous skin lesions from the mouth and periorbital regions of the left and right eyes shows the evidence of gross, well-circumscribed, popular, crusting lesions. Histological examinations of the skin demonstrated the focal to diffuse full-thickness necrosis of the epidermis and a thick serocellular surface crust (Fig. S1A). All skin nodules were composed of a massive proliferation of keratinocytes containing eosinophilic intracytoplasmic inclusions that were consistent with an avian poxvirus infection (Fig. S1B). There was a variable infiltration of the dermis with mixed inflammatory cells, being heterophilic in some areas and lymphocytic in others. In some areas, there were also hemorrhage and serocellular encrustation on the surface. Throughout the liver, there was a mild accumulation of brownish-golden intracytoplasmic pigment granules in Kupffer cells and hepatocytes, which were likely haemosiderin. The kidney and pancreas appeared histologically normal.

### Evidence of poxvirus particles in cutaneous pox lesions.

**FIG 1:**
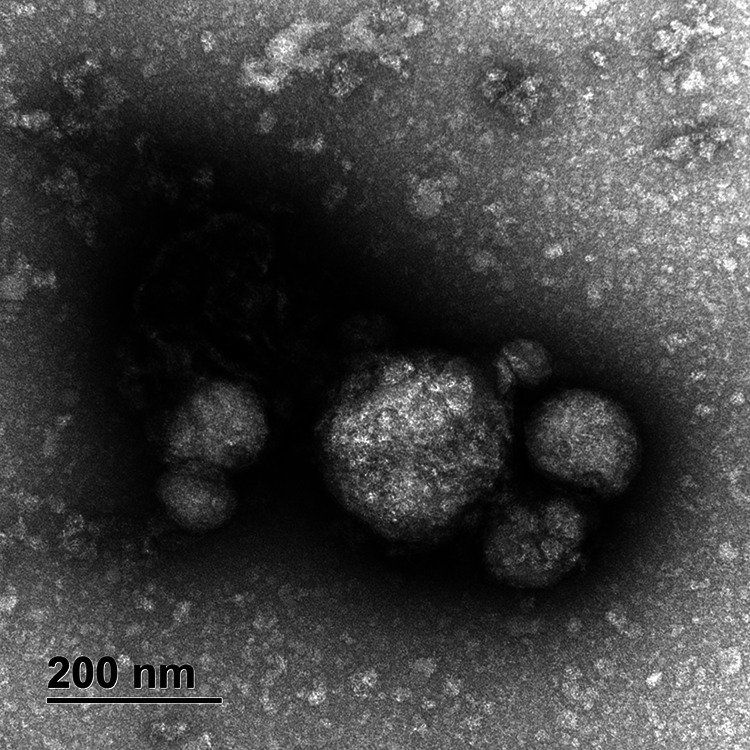
Transmission electron microscopic analysis of negatively stained cutaneous tissue sourced from Cook’s petrel.

A transmission electron microscopic analysis of affected cutaneous tissues clearly identified poxvirus virus particles (Fig. 1) that were indicative of an active poxvirus infection in the Cook’s petrel. According to Harrion et al. (24), one stage of virus particles, such as immature virions (IV) (Fig. 1), was detected in the affected tissue collected from the Cook’s petrel. Morphologically, the immature virions were rectangular or ovoid shaped with rounded corners (Fig. 1). The immature virions had a length of approximately 90 to 240 nm and a width of 60 to 200 nm.

### Genome of Cook’s petrelpox virus (CPPV).

The complete genome sequence of CPPV was determined to be a linear double-stranded DNA molecule that was 314,065 bp in length (GenBank accession no. OP292971). The CPPV genome contained a large central coding region that was surrounded by two matching inverted terminal repeat (ITR) regions, each constituted of 3,552 bp (coordinates 1 to 3,552 sense and 310,544 to 314,065 antisense orientation), like other characterized avipoxviruses (11, 17, 19, 25, 26). The novel CPPV genome showed the highest nucleotide identity with a pathogenic avipoxvirus FGPV (88.9%) that was recently isolated from lesser flamingos (Phoenicopterus minor) in South Africa in 2008 (GenBank accession no. MF678796) (21), and this was followed by PEPV (84.7%), FeP2 (81.4), ALPV2 (74.7%), and FWPV (74.5%) (Table 1). The A+T content of the CPPV genome was 71.3%, which is consistent with the range of the A+T content of other complete genomes of avipoxviruses (Table 1).

**TABLE 1:** Comparative analysis of representative avipoxviruses and CPPV, based on complete genome nucleotide sequences

Avipoxviruses (abbreviation)	GenBank accession no.	Genome identity (%)	Genome length (kbp)	A + T content (%)	No. of ORFs	Reference
Cook’s petrelpox virus (CPPV)	OP292971		314	71.3	357	This study
Crowpox virus (CRPV)	ON408417	51.6	329	71.3	403	56
Albatrosspox virus 2 (ALPV2)	OK348853	74.7	286	69.1	359	8
Albatrosspox virus (ALPV)	MW365933	50.1	352	71.2	336	9
Canarypox virus (CNPV)	AY318871	46.8	360	69.6	328	28
Fowlpox virus (FWPV)	AF198100	74.5	289	69.1	260	25
Flamingopox virus (FGPV)	MF678796	88.9	293	70.5	285	21
Finch poxvirus (FIPV)	OM869483	48.4	354	69.9	334	20
Magpiepox virus (MPPV)	MK903864	51.9	293	70.4	301	17
Magpiepox virus 2 (MPPV2)	MW485973	52.2	298	70.5	419	18
Mudlarkpox virus (MLPV)	MT978051	49.6	343	70.2	352	19
Penguinpox virus (PEPV)	KJ859677	84.7	307	70.5	285	27
Penguinpox virus 2 (PEPV2)	MW296038	50.3	350	69.9	327	10
Pigeonpox virus (FeP2)	KJ801920	81.4	282	70.5	271	27
Shearwaterpox virus 1 (SWPV1)	KX857216	52.8	327	72.4	310	11
Shearwaterpox virus 2 (SWPV2)	KX857215	49.6	351	69.8	312	11
Chelonid poxvirus 1 (ChPV1)	MT799800	49.6	343	71.6	329	30
Turkeypox virus (TKPV)	KP728110	37.3	189	70.2	171	57

### Genome annotation and comparative analyses of CPPV.

**FIG 2:**
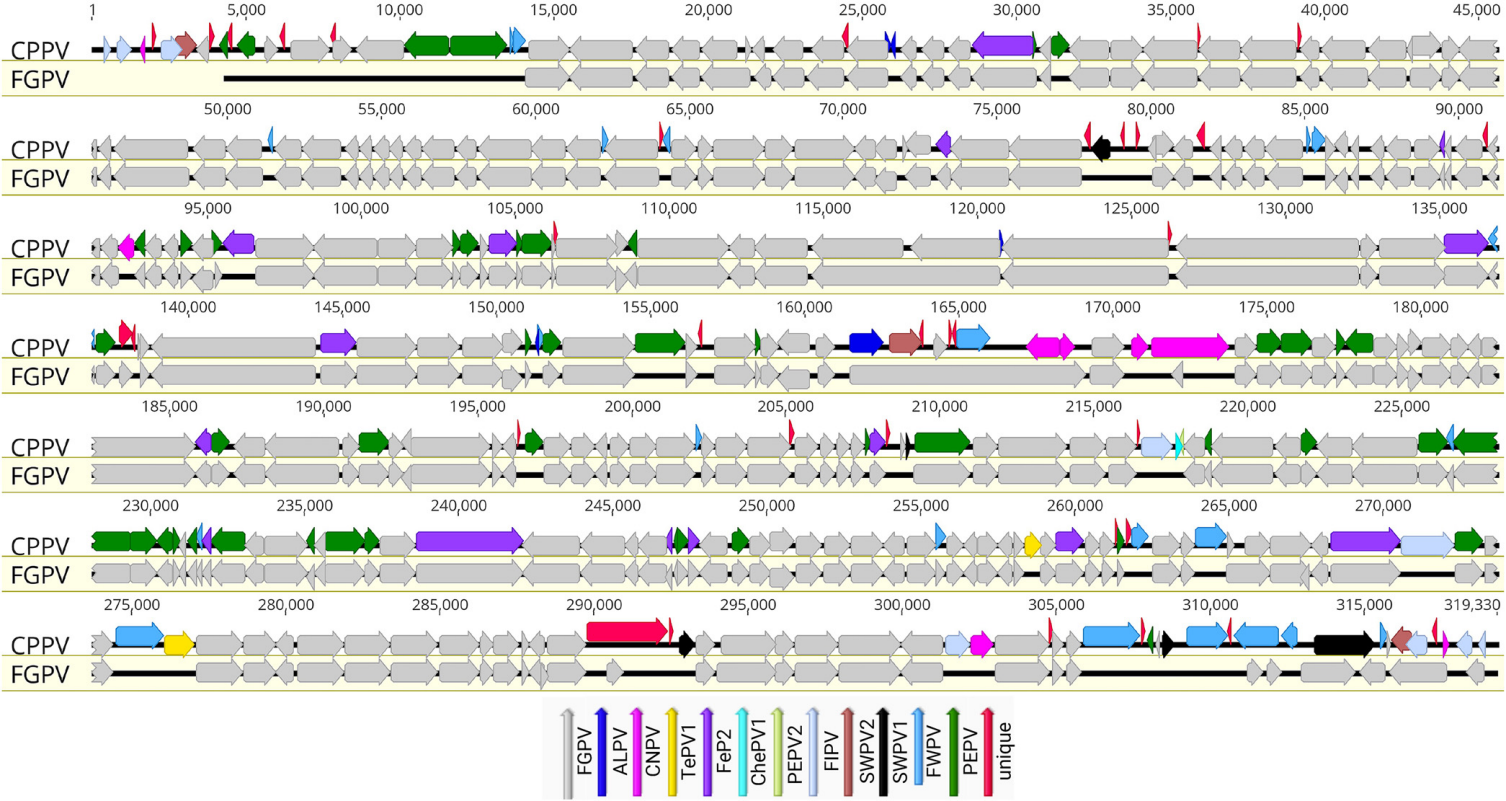
Genomic illustration of CPPV in comparison with FGPV, visualized using Geneious Prime (version 2022.1.1). The arrows depict the direction of the transcription of genes and open reading frames (ORFs). Each ORF of the CPPV genome is color coded, based on its homology to other avipoxviruses, as indicated by the key in the legend.

The CPPV genome was predicted to enclose 357 open reading frames (ORFs) encoding proteins ranging from 30 to 1,969 amino acids in length, and they were numbered from left to right (Fig. 2; Table S1). Among them, six predicted ORFs were found within the ITRs and were thus present as diploid copies. A comparative analysis of the predicted ORF sequences showed that 323 had the greatest similarity with other ChPV gene products (E value of ≤10^−5^) (Fig. 2; Table S1). Among these predicted genes, the highest number of genes (*n* = 206) showed the highest similarity to FGPV (21), and this was followed by 46 genes to PEPV (15) and 22 genes to FWPV (25). A further 15 genes (CPPV-038, -086, -109, -122, -130, -149, -157, -200, -229, -258, -267, -272, -275, -293, and -307) showed the highest similarity to FeP2 (27), 9 genes (CPPV-001, -002, -005, -239, -308, -334, -353, -356, and -357) to a recently sequenced FIPV (20), 8 genes (CPPV-003, -115, -182, -183, -185, -186, -335, and -355) to CNPV (28), 5 genes (CPPV-033, -034, -143, -163, and -175) to ALPV (9), 5 genes (CPPV-090, -232, -327, -344, and -349) to SWPV1 (11), 3 genes (CPPV-006, -176, and -352) to SWPV2 (11), 2 genes (CPPV-291 and -312) to TePV1 (29), 1 gene (CPPV-240) to ChePV1 (30), and 1 gene (CPPV-241) to PEPV2 (10) (Fig. 2; Table S1).

Remarkably, the CPPV genome contained 34 predicted protein-coding genes that were unique, based on the non-redundant (NR) protein database using BLASTX and BLASTP (31). These unique ORFs encoded proteins of 30 to 150 amino acids in length (Fig. 2; Table S1). Among them, 12 unique CPPV protein-coding ORFs (ORF-004, -008, -015, -048, -075, -089, -096, -145, -153, -169, -346, and -354) were predicted to contain a single transmembrane helix (Table S1). We did not find any significant homology with known proteins for the unique ORFs that were encoded in the CPPV genome when using Phyre2, HHpred, or SWISS-MODEL, which may result from the lack of closely related structures in these databases.

Dot plot analyses were used to compare the CPPV genome with other selected avipoxviruses. The CPPV genome was highly syntenic with FGPV, PEPV, ALPV2, and FWPV (Fig. S2A–D); however, a difference in synteny was observed (Fig. S2A–D, highlighted as black arrows), mainly due to the absence of two additional copies of the N1R/p28-like protein gene, two additional copies of the TGF-beta-like protein gene, and two hypothetical protein coding genes covering approximately 6.5 kbp. However, the CPPV genome demonstrated significant differences in the entire genome, compared to other complete avipoxviruses, including CNPV, SWPV2, and TKPV (Fig. S2E–G).

### Core/conserved ORFs.

Similar to other chordopoxviruses (ChPVs), the CPPV genome contained 87 conserved core genes, and these are involved in essential functions, such as replication, transcription, and virion assembly (Table S1, highlighted with bold font). The number of conserved ChPV genes is considered to range between 83 and 90 (21, 27, 32, 33), which is consistent with the findings in the CPPV genome. Among them, five of the predicted ORFs (CPPV-138, -206, -245, -252, and -254) were truncated, mostly with a single residue, compared to a closely related avipoxvirus (FGPV), which may warrant further studies to determine whether they are expressed and functional. Based on a recent study by Carulei et al. (21), we also searched for an additional 47 genes that are conserved in all complete genomes of avipoxviruses (Table S2). The novel CPPV genome also contained these 47 conserved ORFs (Table S2), and nine of the genes (CPPV-036, -043, -057, -074, -077, -083, -129, -282, and -293) were found to be truncated, compared to a closely related flamingopox virus (FGPV).

### Multigene families.

Avipoxviruses are the largest ChPVs, and they contain several, large, multigene families with immune related functions that comprise up to 50% of the genome (21, 27). The copy number of each of the 14 multigene families identified in the CPPV genome was compared with the other selected sequenced avian poxvirus genomes, including the recently characterized genomes of CRPV, ALPV2, ALPV, MPPV2, and PEPV2 (Table S3). CPPV has a relatively higher number of multigene families (124 gene copies), compared to the closely related avipoxviruses, such as FGPV, FeP2, and PEPV (a total of 103, 67, and 80 gene copies, respectively). The copy number of ankyrin repeat, NiR/p28, CC chemokine, and C-type lectin family genes were relatively higher in the CPPV genome, compared to FGPV. However, the copy number of the Ig-like domain gene was significantly lower in the CPPV genome, compared to that of FGPV.

### Evolutionary relationships of CPPV.

**FIG 3:**
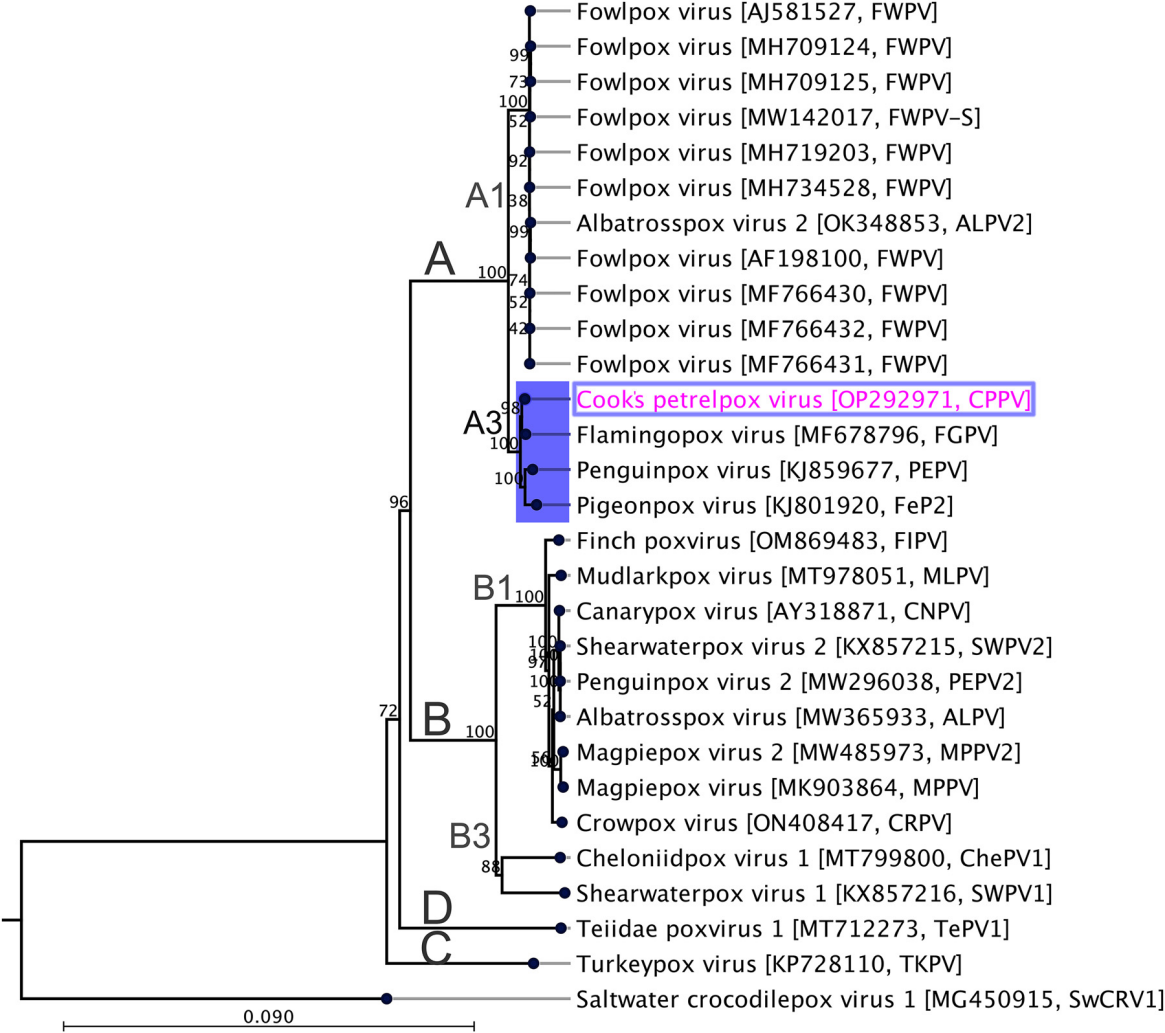
Phylogenetic relationships between CPPV and other chordopoxviruses. A maximum likelihood (ML) tree was constructed from multiple alignments of the concatenated amino acid sequences of the selected nine poxvirus core proteins using CLC Genomics Workbench (version 9.0.1). The numbers on the left show bootstrap values as percentages. The labels at branch tips refer to virus species, and they are followed by the GenBank accession numbers and the abbreviated species names in parentheses. The position of CPPV is highlighted using pink text with a blue box. The details of the poxviruses used in the phylogenetic tree are provided in Table 2. Saltwater crocodile poxvirus 1 (SwCRV1; MG450915) (55) was used as an outgroup. Major clades and subclades are designated according to Gyuranecz et al. (2013) (34).

Phylogenetic reconstruction using the concatenated amino acid sequences of selected conserved ChPV genes provides strong evidence for the inclusion of CPPV in the genus *Avipoxvirus.* In the maximum likelihood (ML) tree (Fig. 3), CPPV was located within subclade A3, which encompasses avipoxviruses that were isolated from South African bird species, such as the lesser flamingo (*Phoenicopterus minor*) (21), feral pigeon (Columba livia), and African penguin (Spheniscus demersus) (27), with 100% bootstrap support. In the subclade A3, CPPV and FGPV are in the same lineage, with strong bootstrap support (98%), suggesting that both of the avipoxviruses might have evolved from a possible Gondwanan ancestor, given that the extant range for Cook’s petrel is restricted to throughout the Pacific Ocean. Using the same set of concatenated protein sequences, we found that the maximum interlineage sequence identity values ranged from 98.5% to 99.5% among avipoxviruses under subclade A3, which further supports the phylogenetic congruence of this novel avipoxvirus that was sequenced from a vulnerable cook’s petrel and further implies that these viruses likely originated from a common progenitor. Additionally, it was revealed that there are many avipoxviruses that are evolutionarily linked with the Cook’s petrelpox virus that was sequenced in this study, compared by using partial nucleotide sequences of the DNA polymerase gene (Fig. S3) and the p4b gene (Fig. S4). In agreement with the genomic level identities, genome architecture, and evolutionary relationship based on concatenated sequences, we also found that avipoxviruses isolated from South African bird species, such as the lesser flamingo (*Phoenicopterus minor*) (21), feral pigeon (*Columba livia*), and African penguin (*Spheniscus demersus*) (27), were the closest evolutionary links with the CPPV that was isolated in this study (Fig. S3 and S4). In addition, many other avipoxviruses, including one from a Southern giant petrel (Macronectes giganteus) from Antarctica, a rock dove (Columba livia) from North America, a great bustard (Otis tarda) from Spain, an oriental turtle-dove (Streptopelia orientalis), and a Eurasian eagle owl (Bubo bubo) from South Korea (34) (Fig. S3 and S4), were clustered within the same clade as CPPV, and they were also shown to be almost identical to CPPV within the relatively small fragment of p4b and DNA polymerase genes.

## DISCUSSION

This study represents evidence of a novel pathogenic avipoxvirus, namely, Cook’s petrelpox virus (CPPV), in the vulnerable Cook’s petrel (*Pterodroma cookii*) in Australia. Surprisingly, given that the range of Cook’s petrels is restricted to the Pacific Ocean, the novel CPPV showed the highest, closest genetic relationship with an avipoxvirus that was isolated from a South African bird, namely, a lesser flamingo (*Phoenicopterus minor*) (21), after comparing genome level identity, gene content, and overall genomic architecture. However, CPPV was distinct from other avipoxvirus genomes in multiple ways. Overall, the DNA sequence of CPPV was significantly different from other avipoxviruses but had the closest similarity with a pathogenic avipoxvirus, namely, a recently isolated FGPV (88.9%) from a lesser flamingo (*Phoenicopterus minor*) in South Africa (21), and this was followed by PEPV (84.7%), FeP2 (81.4), ALPV2 (74.7%), and FWPV (74.5%). The novel CPPV genome contained 34 predicted genes that are not found in any other poxvirus as well as several ORFs that were truncated/fragmented so as to probably cause them to be nonfunctional. Overall, the CPPV was sufficiently genetically different to other previously classified avipoxviruses so as to be considered a distinct, new virus species under the genus *Avipoxvirus*.

This study represents an avipoxvirus that was phylogenetically located in the subclade A3 (Fig. 3; Fig. S3 and S4). The subclade A3 consists of avipoxviruses that were isolated from several bird species worldwide, including this Cook’s petrel as well as several South African birds, such as a lesser flamingo (*Phoenicopterus minor*) (21), a feral pigeon (*Columba livia*), an African penguin (*Spheniscus demersus*) (27), and many others from the USA, Spain, and South Korea. In comparison with partial p4b and DNA polymerase genes, our CPPV sequence clustered with many other avipoxviruses, including an avipoxvirus from a Southern giant petrel (Macronectes giganteus) from Antarctica, which are distantly related to the Cook’s petrel (*Pterodroma cookii*) (35) but share ecological niches. This may suggest that all of these viruses in subclade A3 (Fig. 3) may have emerged from a possible Gondwanan ancestor. A shallow viral host switch event from an unknown host is also a possibility, given the lack of knowledge of avipoxvirus ecology as well as recent reports of avipoxvirus-like viruses in reptiles, such as the green sea turtle (Chelonia mydas) (30) and crocodile tegu (Crocodilurus amazonicus) (29). Several recent papers report evidence of avipoxviruses in other seabirds in Australia and New Zealand (8–11), and it is possible that many of these species may harbor other, yet to be discovered avipoxviruses.

Avian pox is usually an endemic, mild, and self-limiting disease among wild birds, but epizootics among endemic birds on remote islands, such as the Hawaiian Islands (12), Galapagos Archipelago (7), Canary Islands (36), and Falkland Islands (23) have been characterized by high morbidity and mortality. However, much is still unknown about the host spectrum, virulence, and transmission dynamics of poxviruses for Australasian animal hosts. Several independent studies suggest that avipoxviruses can be transmitted between birds in several ways: (i) via direct contact with infected birds through broken skin; (ii) via contaminated objects, such as perches; (iii) via aerosol transmission (22, 37), and (iv) via haematophagous arthropods, including mosquitoes, which are efficient mechanical vectors through contaminated mouthparts (38, 39). Importantly, a recent study reported the evidence of a high frequency of DNA of avipoxviruses in *Culex* spp. that were captured in domestic areas, and it also showed the likely transmission dynamics of APVs in backyard poultry in Rio de Janeiro (40). These may indicate a potential scenario for the transmission of Cook’s petrelpox virus that merits further attention. In addition, at an individual level, poxvirus infections in wild birds may lead to secondary bacterial or fungal infections, a reduction of the ability to care for young, and effects to vision and/or feeding ability, making them prone to predation and significantly affecting welfare (15, 41, 42). The repeated occurrence of avian family or order-specific grouping within certain avipoxvirus clades indicates a marked role of host adaptation, while the sharing of poxvirus species withing prey-predator systems (e.g., pigeons and raptors) (34) indicates the potential for cross-species infection and limited host adaptation (34). At a population level, these may have serious implications, especially for endangered or endemic species. Hence, the evolution of avipoxviruses in nonmodel hosts warrants further investigation.

### Conclusions.

The novel pathogenic avipoxvirus of CPPV that is reported here has enriched our understanding of the avipoxviruses, more generally, and the tracking of poxvirus evolution in a nonmodel, vulnerable seabird species. By assessing the sequence similarity between CPPV and other avipoxviruses and by following these assessments with TEM images of poxvirus particles and evolutionary links, we concluded that the CPPV complete genome that is described here should be considered to be a separate avipoxvirus species, tentatively designated Cook’s petrelpox virus, under the genus *Avipoxvirus*. Additional investigations will be required to better understand relevant host-pathogen dynamics, including routes of transmission, factors leading to infection, associated pathology, and disease prevalence.

## MATERIALS AND METHODS

### Sampling, ethical consideration, and extraction of DNA.

An alcohol-preserved cutaneous tissue from the mouth and periorbital regions of the left and right eyes with characteristic pox lesions from a Cook’s petrel (*Pterodroma cookii*) was collected at Brisbane Bird and Exotics Veterinary Service, QLD-4120, by the attending veterinarian, and it was sent to the Veterinary Diagnostic Laboratory, Charles Sturt University, for analysis (sample ID: 22-0390; year of sampling: 2022). The sample was from a bird that died from unrelated injuries soon after being admitted for veterinary care and was found after a storm in Southern Queensland. After an initial histopathological examination, the sample was sent to La Trobe University for molecular investigation. Animal sampling was obtained by following approved guidelines set by the Australian Code of Practice for the Care and Use of Animals for Scientific Purposes (1997) and was approved by the Charles Sturt University Animal Ethics Committee (Research Authority permit 09/046). The crusty pox lesion material was aseptically dissected and mechanically homogenized in lysis buffer using disposable tissue grinder pestles, and it was then transferred into a 1.5 mL microcentrifuge tube (Eppendorf). Total genomic DNA was isolated according to the established methods (43–45) using a ReliaPrep gDNA Tissue Miniprep System (Promega, USA).

### Library construction and sequencing.

A total of 250 ng of extracted genomic DNA was used to prepare the library, using the adapted previously protocol for the Illumina DNA Prep (Illumina, San Diego, CA, USA) (46). The quality and quantity of the prepared library were assessed using an Agilent Tape Station (Agilent Technologies) by the Genomic Platform, La Trobe University. The prepared library was sequenced with a sequencing reads length of 150 bp paired-ends on an Illumina NovaSeq platform, according to the manufacturer’s instructions, through the Australian Genome Research Facility, Melbourne.

### Genome assembly and annotation.

The resulting 33.9 million raw sequence reads were used to assemble the complete genome of CPPV, using CLC Genomics Workbench (version 9.0.1, CLC bio, a Qiagen Company, Prismet, Aarhus C, Denmark) and Geneious Prime (version 2022.1.1, Biomatters, New Zealand), as described previously (10, 11, 17, 26, 47). Briefly, the sequences were processed to remove Illumina adapters, low quality reads, and ambiguous bases. Trimmed sequence reads were mapped against the chicken genome (Gallus gallus, GenBank accession number NC_006088.5) to remove potential host DNA contamination. In addition, reads were further mapped to the Escherichia coli bacterial genomic sequence (GenBank accession no. U00096) to remove possible bacterial contamination. A total of 30.2 million cleaned and unmapped reads were used as input data for *de novo* assembly using CLC Genomics Workbench (version 9.0.1). This resulted in the generation of a 314,065 bp genome with an average coverage of 9232.42×. The genome was annotated, according to the previously published protocol using Geneious software (version 2022.1.1). Open reading frames (ORFs) longer than 30 amino acids, with a methionine start codon (ATG) and minimal overlap of other ORFs (not exceeding 50% of one of the genes), were selected and annotated. Similarity BLAST searches were performed on the predicted ORFs, which were annotated as potential genes if predicted the ORFs showed significant sequence similarity to known viral or cellular genes (BLAST E value of ≤*e*^−5^) (31).

To predict the functions of the putative unique ORFs identified in this study, the derived protein sequence of each ORF was searched using multiple applications to identify conserved domains or motifs. Transmembrane helices were searched using the HMMTOP (48) and TMpred (49). Additionally, searches for conserved secondary structure (HHpred) (50) and protein homologs were conducted using Phyre2 (51) and SWISS-MODEL (52).

### Comparative genomics.

Genomic features of the newly sequenced CPPV were visualized using Geneious Prime (version 2022.1.1). The sequence similarity percentages between CPPV and representative ChPV complete genome sequences were determined using tools available in Geneious (version 2022.1.1). Dot plots were created based on the EMBOSS dottup program in Geneious software with word size = 12 (53).

### Phylogenetic analyses.

Phylogenetic analyses were performed using the CPPV genome sequence that was determined in this study together with other selected ChPV genome sequences that are available in GenBank (Table 2). The amino acid sequences of nine poxvirus core proteins (RNA polymerase subunit RPO132, RNA polymerase subunit RPO147, mRNA capping enzyme large subunit, RNA polymerase-associated protein RAP94, virion core protein P4a, virion core protein P4b, early transcription factor large subunit VETFL, NTPase, and DNA polymerase) were concatenated and aligned using MAFTT (version 7.450) with the G-INS-i (gap open penalty of 1.53; offset value of 0.123) were algorithm implemented in Geneious Prime (version 2022.1.1, Biomatters, New Zealand). Nucleotide sequences of the partial DNA polymerase and partial p4b genes, as well as concatenated amino acid sequences of the selected nine poxvirus core proteins, were aligned, as described previously (19), using the MAFTT L-INS-I algorithm, implemented in Geneious Prime (version 2022.1.1) (version 7.388) (54). To determine the best fit model with which to construct the phylogenetic analyses, a model test was performed using CLC Genomics Workbench (version 9.0.1), which favored a general-time-reversible model with a gamma distribution rate variation and a proportion of invariable sites (GTR+G+I). The phylogenetic analyses for the nucleotide sequences were performed under the GTR substitution model, but the WAG substitution model was chosen for the concatenated amino acid sequences, using 1,000 bootstrap replicates in CLC Genomic Workbench (version 9.0.1).

**TABLE 2:** Related poxvirus genome sequences used in further analysis of CPPV

Virus	Abbreviation	Year of isolation	GenBank accession no.	Reference
Albatrosspox virus 2	ALPV2	1997	OK348853	8
Albatrosspox virus	ALPV	1997	MW365933	9
Canarypox virus	CNPV	1948	AY318871	28
Canarypox virus	CNPV	2015	MG760432	58
Cook’s petrelpox virus	CPPV	2022	OP292971	This study
Crowpox virus	CRPV	2021	ON408417	56
Cheloniidpox virus 1	ChePV1	2018	MT799800	30
Fowlpox virus	FWPV	2012, 2000^*a*^, 2010^*a*^, 2015, 2016, 2018^*a*^, 2011^*b*^, 2018	MW142017, AF198100^*a*^, AJ581527^*a*^, MH734528, MH719203, MF766430-32, MH709124-25^*a*^, MG702259^*b*^, OK558608-09	25, 59–62
Flamingopox virus	FGPV	2008	MF678796	21
Finch poxvirus	FIPV	2021	OM869483	20
Magpiepox virus	MPPV	2018	MK903864	17
Magpiepox virus 2	MPPV2	1956	MW485973	18
Mudlarkpox virus	MLPV	2019	MT978051	19
Penguinpox virus	PEPV	1992	KJ859677	27
Penguinpox virus 2	PEPV2	1997	MW296038	10
Pigeonpox virus	FeP2	1992	KJ801920	27
Saltwater crocodilepox virus 1	SwCRV1	2017	MG450915	45, 55
Shearwaterpox virus 1	SWPV1	2015	KX857216	11
Shearwaterpox virus 2	SWPV2	2015	KX857215	11
Turkeypox virus	TKPV	2011	NC_028238	57
Teiidae poxvirus 1	TePV1	2019	MT712273	29

a The year of submission to GenBank is reported.

b Unpublished.

### Transmission electron microscopy.

Cutaneous pox lesions were suspended 1:10 in phosphate-buffered saline (PBS), homogenized, clarified, and adsorbed onto 400-mesh copper EM grids before staining and imaging on a JEOL JEM-2100 transmission electron microscope, as previously described (26, 55).

### Data availability.

The complete genome sequence and the associated data sets that were generated during this study were deposited in GenBank under the accession number OP292971.
