# Supplementary material for: A Novel Pathogenic Avipoxvirus Infecting Vulnerable Cook’s Petrel (Pterodroma cookii) in Australia Demonstrates a High Genomic and Evolutionary Proximity with South African Avipoxviruses
Source: Microbiol Spectr. 2023 Feb 7;11(2):e04610-22. doi: 10.1128/spectrum.04610-22 (PMC10100368; doi:10.1128/spectrum.04610-22)
Supplement: Supplemental file 1 — Supplemental material. Download spectrum.04610-22-s0001.pdf, PDF file, 2.8 MB [file spectrum.04610-22-s0001.pdf]

Supplementary File

A novel pathogenic avipoxvirus infecting vulnerable Cook's petrel (*Pterodroma cookii*) in Australia demonstrates a high genomic and evolutionary proximity with South African avipoxviruses

Subir Sarker [a*] and Shane R. Raidal [b]

[a]Department of Microbiology, Anatomy, Physiology and Pharmacology, School of Agriculture, Biomedicine and Environment, La Trobe University, Melbourne, VIC 3086, Australia.
[b]School of Agricultural, Environmental and Veterinary Sciences, Faculty of Science and Health, Charles Sturt University, Wagga, New South Wales 2678, Australia

*Address for correspondence: Dr. Subir Sarker, Department of Microbiology, Anatomy, Physiology and Pharmacology, School of Agriculture, Biomedicine and Environment, La Trobe University, Melbourne, VIC 3086, Australia; email: S.Sarker@latrobe.edu.au; phone: +61 3 9479 2317; fax: +61 3 9479 1222.

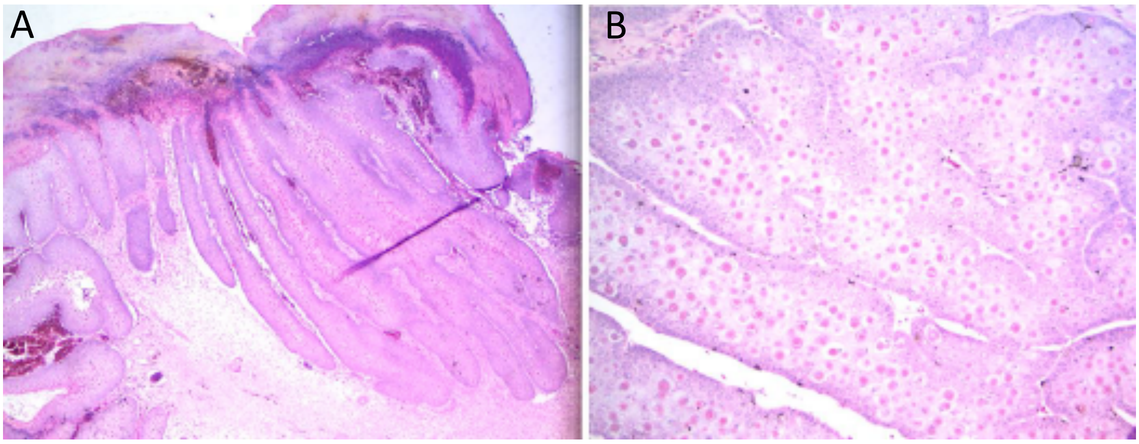

FIG. S1 Pathological evidence of characteristic pox lesions. Skin nodules demonstrating massive proliferation of keratinocytes containing eosinophilic intracytoplasmic inclusions consistent with avian poxvirus infection.

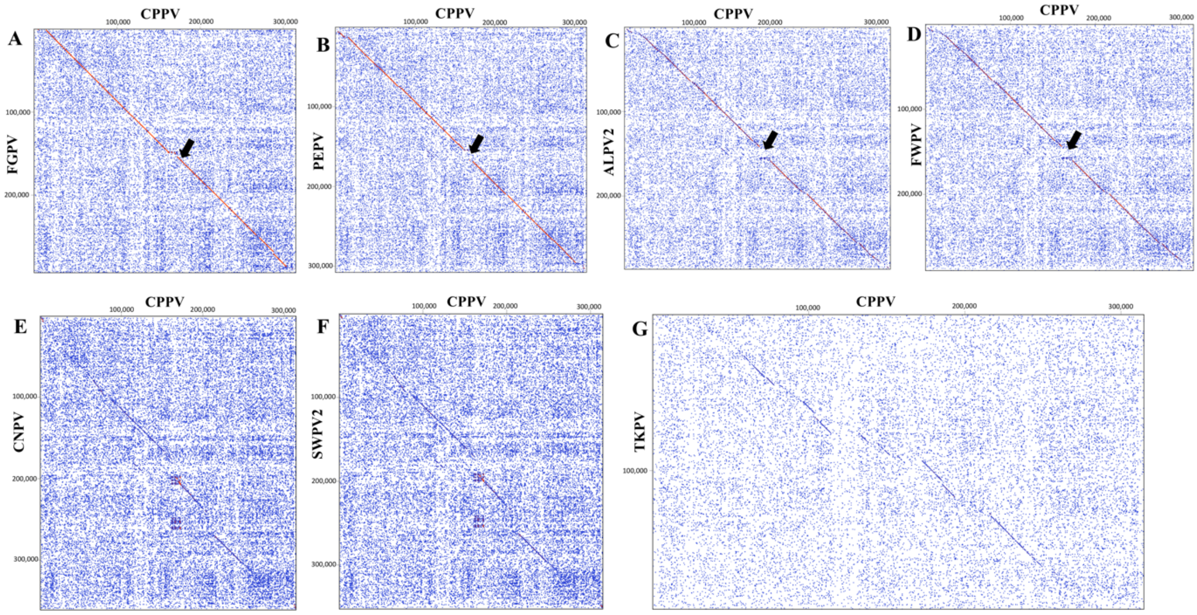

FIG. S2 Dot plots of the CPPV genome (x-axis) versus other poxvirus genomes (y-axis). (A) CPPV vs FGPV, (B) CPPV vs PEPV, (C) CPPV vs ALPV2, (D) CPPV vs FWPV, (E) CPPV vs CNPV, (F) CPPV vs SWPV2 and (G) CPPV vs TKPV (refer to **Table 2** for virus details and GenBank accession numbers). The Classic colour scheme was chosen in Geneious (version 22.1.1) for the dot plot lines according to the length of the match, from blue for short matches to red for matches over 100 bp long. Window size = 12.

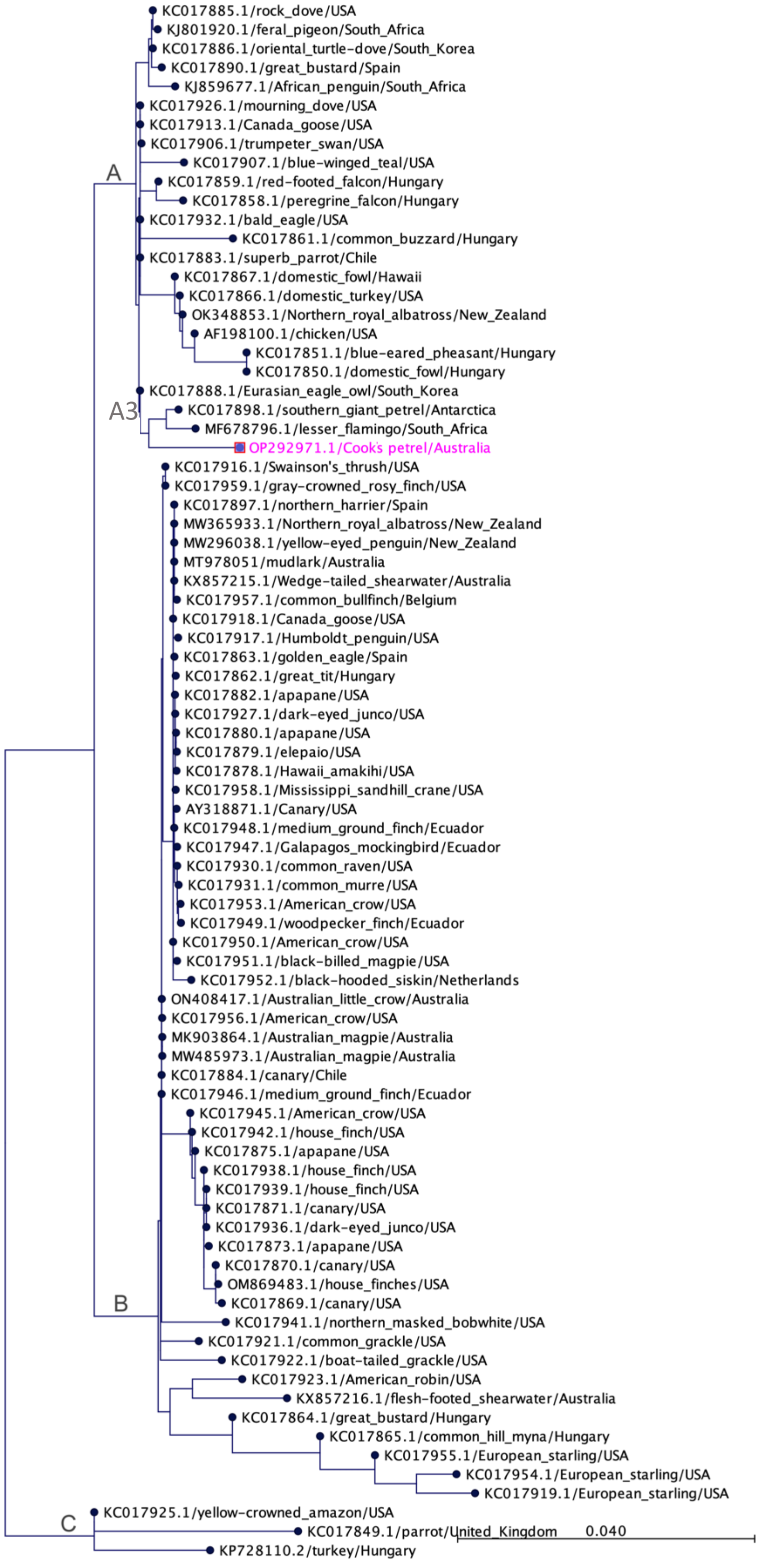

FIG. S3 Maximum likelihood (ML) phylogenetic tree from partial nucleotide sequences of the DNA polymerase gene of selected avipoxviruses. Labels at branch tips refer to GenBank accession number/species/country of origin. The numbers on the left show bootstrap values as percentages. The position of CPPV is highlighted using pink text. The ML tree is displayed as a phylogram. The bootstrap value assigned to a node in the output tree is the percentage (0-100) of the bootstrap resamples which resulted in a tree containing the same subtree as that rooted at the node. Major clades and sub-clades are designated according to Gyuranecz *et al* (2013).

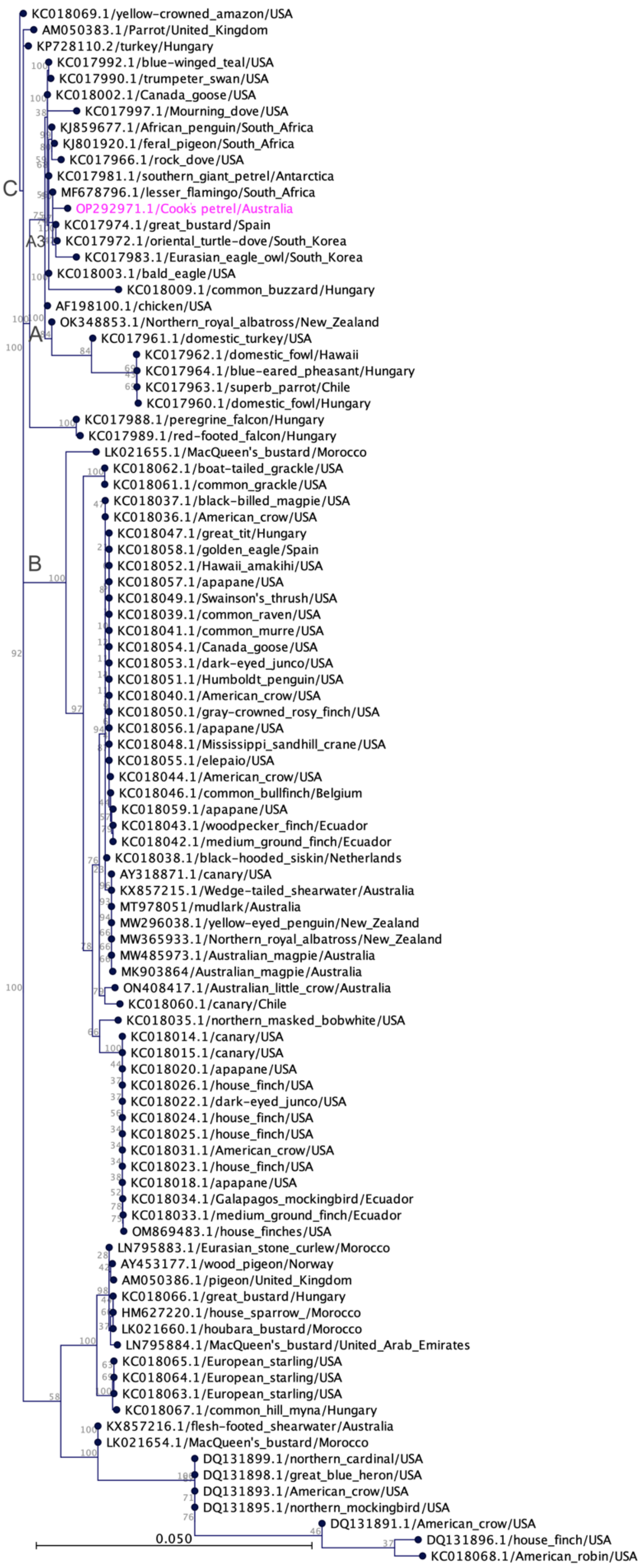

FIG S4 Maximum likelihood (ML) phylogenetic tree from partial nucleotide sequences of the P4b gene of selected avipoxviruses. Labels at branch tips refer to GenBank accession number/species/country of origin. The numbers on the left show bootstrap values as percentages. The position of CPPV is highlighted using pink text. The ML tree is displayed as a phylogram. The bootstrap value assigned to a node in the output tree is the percentage (0-100) of the bootstrap resamples which resulted in a tree containing the same subtree as that rooted at the node. Major clades and sub-clades are designated according to Gyuranecz *et al* (2013).

Supplementary Table S1. Crowpox virus (CPPV) genome annotations and comparative analysis of ORFs

CPPV Synteny	CPPV Genome Coordinates	FGPV Synteny	FGPV AA size	CPPV AA size	Best BLAST hits	CPPV AA Identity (%) compared to avipoxviruses	notes
CPPV001	401-619			72	hypothetical protein [FIPV]	83.9%	
CPPV002	824-1300			158	hypothetical protein [FIPV]	96.2%	
CPPV003	1725-1570			51	C-type lectin-like protein [CNPV]	88.2%	
CPPV004	1948-2058			36			hypothetical protein, unique to CPPV, containing a transmembrane helix
CPPV005	2245-2913			222	hypothetical protein [FIPV]	97.3%	
CPPV006	2732-3376			214	conserved hypothetical protein [SWPV2]	93.2%	
CPPV007	3775-3401			124	C-type lectin family protein [FGPV]	97.1%	
CPPV008	3830-3955			41			hypothetical protein, unique to CPPV, containing a transmembrane helix
CPPV009	4405-4154			83	C-type lectin-like protein [PEPV]	82.5%	
CPPV010	4539-4396			47			hypothetical protein, unique to CPPV
CPPV011	5286-4711			191	ankyrin repeat protein [PEPV]	80.6%	
CPPV012	5620-6042			140	hypothetical protein [FGPV]	100.0%	
CPPV013	6241-6107			44			hypothetical protein, unique to CPPV
CPPV014	6478-7734			418	C4L/C10L family protein [FGPV]	99.0%	

**CPPV Synteny**	**CPPV Genome Coordinates**	**FGPV Synteny**	**FGPV AA size**	**CPPV AA size**	**Best BLAST hits**	**CPPV AA Identity (%) compared to avipoxviruses**	**notes**
CPPV015	7880-7737			47			hypothetical protein, unique to CPPV, containing a transmembrane helix
CPPV016	7822-8427			201	hypothetical protein [FGPV]	98.5%	
CPPV017	10093-8567			508	ankyrin repeat protein [FGPV]	99.2%	
CPPV018	11559-10144			471	ankyrin repeat protein [PEPV]	36.6%	
CPPV019	11658-13478			606	ankyrin repeat protein [PEPV]	36.0%	
CPPV020	13569-13700			43	C-type lectin [FWPV]	80.7%	
CPPV021	13684-14070			128	C-type lectin family protein [FWPV]	65.6%	
CPPV022	14172-15506	FGPV001	474	444	ankyrin repeat protein [FGPV]	99.3%	
CPPV023	17549-15501	FGPV002	682	682	ankyrin repeat protein [FGPV]	98.6%	
CPPV024	18722-17655	FGPV003	355	355	serpin family protein [FGPV]	97.5%	
CPPV025	19704-18823	FGPV004	293	293	alpha-SNAP [FGPV]	99.3%	
CPPV026	20919-19780	FGPV005	520	379	ankyrin repeat protein [FGPV]	98.7%	
CPPV027	21220-21402	FGPV005	520	60	ankyrin repeat protein [FGPV]	100.0%	
CPPV028	21622-21413	FGPV006	174	69	interleukin 10 [FGPV]	98.5%	
CPPV029	22832-21852	FGPV007	330	326	ankyrin repeat protein [FGPV]	98.8%	
CPPV030	24160-23093	FGPV008	402	355	ankyrin repeat protein [FGPV]	99.2%	
CPPV031	24270-24106			54			hypothetical protein, unique to CPPV
CPPV032	25558-24245	FGPV009	437	437	ankyrin repeat protein [FGPV]	99.3%	
CPPV033	25494-25685			63	hypothetical protein [ALPV]	87.8%	
CPPV034	25815-25675			46	hypothetical protein [ALPV]	89.1%	

CPPV Synteny	CPPV Genome Coordinates	FGPV Synteny	FGPV AA size	CPPV AA size	Best BLAST hits	CPPV AA Identity (%) compared to avipoxviruses	notes
CPPV035	26368-25988	FGPV010	171	126	hypothetical protein [FGPV]	70.8%	
CPPV036	27268-26552	FGPV011	239	238	Ig-like domain protein [FGPV]	96.2%	
CPPV037	28118-27381	FGPV012	245	245	V-type Ig domain protein [FGPV]	95.9%	
CPPV038	30125-28194	FGPV013	684	643	ankyrin repeat protein [FeP2]	96.6%	
CPPV039	30133-30234			33	ankyrin repeat protein [PEPV]	90.9%	
CPPV040	30714-30373			113	hypothetical protein [FGPV]	98.2%	
CPPV041	30743-31312	FGPV014	113	189	hypothetical protein [PEPV]	95.2%	
CPPV042	32595-31309	FGPV015	428	428	C4L/C10L protein [FGPV]	99.3%	
CPPV043	32692-33699	FGPV016	337	335	G-protein-coupled receptor family protein [FGPV]	99.4%	
CPPV044	35456-33714	FGPV017	580	580	ankyrin repeat protein [FGPV]	99.3%	
CPPV045	35478-35570			30			hypothetical protein, unique to CPPV
CPPV046	36836-35532	FGPV018	434	434	ankyrin repeat protein [FGPV]	99.3%	
CPPV047	38685-36901	FGPV019	595	594	ankyrin repeat protein [FGPV]	98.5%	
CPPV048	38754-38849			31			hypothetical protein, unique to CPPV, containing a transmembrane helix
CPPV049	39428-38817	FGPV020	203	203	hypothetical protein [FGPV]	98.5%	
CPPV050	40909-39479	FGPV021	498	476	ankyrin repeat protein [FGPV]	97.3%	
CPPV051	42243-41020	FGPV022	406	407	ankyrin repeat protein [FGPV]	99.0%	
CPPV052	42434-42255	FGPV023	333	59	G-protein-coupled receptor family protein [FGPV]	100.0%	

CPPV Synteny	CPPV Genome Coordinates	FGPV Synteny	FGPV AA size	CPPV AA size	Best BLAST hits	CPPV AA Identity (%) compared to avipoxviruses	notes
CPPV053	42405-43376	FGPV023	333	323	G-protein-coupled receptor family protein [FGPV]	98.8%	
CPPV054	43426-43968	FGPV024	180	180	hypothetical protein [FGPV]	98.9%	
CPPV055	45347-43953	FGPV025	464	464	ankyrin repeat protein [FGPV]	100.0%	
CPPV056	45791-45411	FGPV026	126	126	peptidyl-tRNA hydrolase [FGPV]	99.2%	
CPPV057	48321-45898	FGPV027	815	807	alkaline phosphodiesterase [FGPV]	98.4%	
CPPV058	49501-48476	FGPV028	341	341	ankyrin repeat protein [FGPV]	99.7%	
CPPV059	50688-49561	FGPV029	382	375	DNase II [FGPV]	99.7%	
CPPV060	51071-50898			57	hypothetical protein [FWPV]	83.0%	
CPPV061	51999-51124	FGPV030	291	291	alpha-SNAP [FGPV]	100.0%	
CPPV062	53256-52021	FGPV031	411	411	ankyrin repeat protein [FGPV]	99.0%	
CPPV063	53835-53428	FGPV032	135	135	hypothetical protein [FGPV]	99.3%	
CPPV064	54281-53874	FGPV033	131	135	hypothetical protein [FGPV]	89.6%	
CPPV065	54852-54358	FGPV034	164	164	hypothetical protein[FGPV]	97.6%	
CPPV066	55286-54849	FGPV035	145	145	dUTP pyrophosphatase [FGPV]	100.0%	
CPPV067	55865-55338	FGPV036	175	175	B-cell lymphoma 2 [FGPV]	99.4%	
CPPV068	56934-55921	FGPV037	337	337	serpin family protein [FGPV]	98.2%	
CPPV069	57649-57014	FGPV038	211	211	hypothetical protein [FGPV]	98.1%	
CPPV070	59430-57736	FGPV039	564	564	DNA ligase [FGPV]	99.7%	
CPPV071	60540-59464	FGPV040	358	358	serpin family protein [FGPV]	99.2%	
CPPV072	61694-60582	FGPV041	368	370	hyroxysteroid dehydrogenase [FGPV]	98.7%	
CPPV073	61776-61937	FGPV042	576	53	semaphorin vaccinia A39R-like protein [FWPV]	65.4%	
CPPV074	63571-61850	FGPV042	576	573	semaphorin [FGPV]	98.1%	

CPPV Synteny	CPPV Genome Coordinates	FGPV Synteny	FGPV AA size	CPPV AA size	Best BLAST hits	CPPV AA Identity (%) compared to avipoxviruses	notes
CPPV075	63631-63750			39			hypothetical protein, unique to CPPV, containing a transmembrane helix
CPPV076	63935-63723			70	hypothetical protein [FWPV]	73.6%	
CPPV077	64036-64815	FGPV043	261	259	GNS1/SUR4 [FGPV]	100.0%	
**CPPV078**	**64892-65356**	**FGPV044**	**154**	**154**	**late transcription factor 2 [FGPV]**	**99.4%**	
**CPPV079**	**65377-67035**	**FGPV045**	**552**	**552**	**rifampicin resistance N3L protein [FGPV]**	**99.8%**	
**CPPV080**	**67067-67936**	**FGPV046**	**289**	**289**	**mRNA capping enzyme small subunit [FGPV]**	**98.3%**	
**CPPV081**	**68017-69930**	**FGPV047**	**637**	**637**	**NPH-1 transcription termination factor [FGPV]**	**99.7%**	
**CPPV082**	**70611-69934**	**FGPV048**	**225**	**225**	**mutT motif [FGPV]**	**98.7%**	
CPPV083	71290-70595	FGPV049	237	231	mutT motif [FGPV]	99.1%	
CPPV084	71506-71673	FGPV050	274	55	V-type Ig domain protein [FGPV]	92.3%	
CPPV085	72407-71592	FGPV050	274	271	V-type Ig domain protein [FGPV]	95.3%	
**CPPV086**	**73060-72575**	**FGPV051**	**161**	**161**	**RNA polymerase subunit RPO18 [FeP2]**	**99.4%**	
**CPPV087**	**74948-73047**	**FGPV052**	**633**	**633**	**early transcription factor small subunit [FGPV]**	**100.0%**	
CPPV088	77304-74929	FGPV053	791	791	NTPase [FGPV]	99.5%	
CPPV089	77492-77388			34			hypothetical protein, unique to CPPV, containing a transmembrane helix
CPPV090	78132-77542	FGPV054	219	196	CC chemokine-like protein [SWPV1]	49.7%	

**CPPV Synteny**	**CPPV Genome Coordinates**	**FGPV Synteny**	**FGPV AA size**	**CPPV AA size**	**Best BLAST hits**	**CPPV AA Identity (%) compared to avipoxviruses**	**notes**
CPPV091	78547-78449			32			hypothetical protein, unique to CPPV
CPPV092	78882-78977			31			hypothetical protein, unique to CPPV
CPPV093	79542-79297	FGPV055	196	81	CC-chemokine family protein [FGPV]	100.0%	
CPPV094	79439-80098	FGPV056	128	219	CC-chemokine family protein [FGPV]	97.7%	
CPPV095	80698-80108	FGPV057	196	196	CC-chemokine family protein [FGPV]	98.0%	
CPPV096	81085-80840			81			hypothetical protein, unique to CPPV, containing a transmembrane helix
CPPV097	81647-81267	FGPV057	196	126	CC-chemokine family protein [FGPV]	89.6%	
CPPV098	82322-81729	FGPV057	196	197	CC-chemokine family protein [FGPV]	93.6%	
**CPPV099**	**83024-82368**	**FGPV058**	**218**	**218**	**uracil DNA glycosylase [FGPV]**	**99.5%**	
CPPV100	84265-83072	FGPV059	378	397	hypothetical protein C1178_gp062 [FGPV]	99.2%	
CPPV101	84397-84525			42	phospholipid hydroperoxide glutathione peroxidase-like proteinue [FWPV]	85.7%	
CPPV102	84598-85002			134	Glutathione peroxidase [FWPV]	94.0%	
CPPV103	85003-85335	FGPV060	110	110	hypothetical protein [FGPV]	100.0%	
CPPV104	85723-85310	FGPV061	137	137	hypothetical protein [FGPV]	100.0%	
CPPV105	86083-85826	FGPV062	85	85	hypothetical protein [FGPV]	98.8%	
CPPV106	86896-86474	FGPV063	130	140	hypothetical protein [FGPV]	78.2%	
**CPPV107**	**87780-86971**	**FGPV064**	**269**	**269**	**HT motif family protein [FGPV]**	**99.3%**	
**CPPV108**	87898-88719	FGPV065	273	273	HT motif family protein [FGPV]	98.5%	

CPPV Synteny	CPPV Genome Coordinates	FGPV Synteny	FGPV AA size	CPPV AA size	Best BLAST hits	CPPV AA Identity (%) compared to avipoxviruses	notes
CPPV109	88866-88735	FGPV066	43	43	hypothetical protein [FeP2]	100.0%	
CPPV110	89103-88870	FGPV067	77	77	ubiquitin family protein [FGPV]	100.0%	
CPPV111	90088-89225	FGPV068	287	287	hypothetical protein [FGPV]	98.6%	
CPPV112	90256-90107			49			hypothetical protein, unique to CPPV
CPPV113	90844-90284	FGPV069	186	186	beta-NGF-like family protein [FGPV]	99.5%	
CPPV114	91420-90866	FGPV070	176	184	IL-18 binding protein [FGPV]	89.1%	
CPPV115	91933-91451	FGPV071	105	160	hypothetical protein [CNPV]	33.9%	
CPPV116	92285-91971			104	hypothetical protein [PEPV]	95.2%	
CPPV117	92852-92289	FGPV072	187	187	N1R/p28-like protein [FGPV]	98.9%	
CPPV118	93364-92921	FGPV073	147	147	beta-NGF-like family protein [FGPV]	94.6%	
**CPPV119**	**93471-93848**	**FGPV074**	**125**	**125**	**glutaredoxin [PEPV]**	**100.0%**	
**CPPV120**	**94498-93821**	**FGPV075**	**225**	**225**	**putative transcriptional elongation factor [FGPV]**	**100.0%**	
**CPPV121**	**94492-94803**	**FGPV076**	**77**	**103**	**hypothetical protein [PEPV]**	**98.8%**	
CPPV122	95838-94831			335	transforming growth factor B [FeP2]	93.4%	
**CPPV123**	**95910-97790**	**FGPV077**	**626**	**626**	**metalloprotease/G1 glycoprotein [FGPV]**	**98.7%**	
**CPPV124**	**99822-97774**	**FGPV078**	**682**	**682**	**DNA/RNA helicase/NPH-11 [FGPV]**	**99.7%**	
**CPPV125**	**99855-101120**	**FGPV079**	**421**	**421**	**virion core peptidase [FGPV]**	**99.5%**	
**CPPV126**	**101123-102295**	**FGPV080**	**390**	**390**	**DNA-binding protein [FGPV]**	**99.7%**	
**CPPV127**	**102296-102541**	**FGPV081**	**81**	**81**	**IMV membrane protein [PEPV]**	**98.8%**	
CPPV128	102551-103102	FGPV082	185	183	thymidine kinase [PEPV]	97.8%	
**CPPV129**	**103170-103445**	**FGPV083**	**91**	**91**	**HT motif family protein [FGPV]**	**97.8%**	
**CPPV130**	**103477-104355**	**FGPV084**	**291**	**292**	**DNA-binding phosphoprotein [FeP2]**	**97.3%**	

CPPV Synteny	CPPV Genome Coordinates	FGPV Synteny	FGPV AA size	CPPV AA size	Best BLAST hits	CPPV AA Identity (%) compared to avipoxviruses	notes
**CPPV131**	**104356-104553**	**FGPV085**	**65**	**65**	**hypothetical protein [PEPV]**	**98.5%**	
**CPPV132**	**104560-105495**	**FGPV086**	**311**	**311**	**virion protein [PEPV]**	**99.7%**	
CPPV133	105496-105600	FGPV087	34	34	MV entry/fusion complex protein [FGPV]	100.0%	
CPPV134	105578-105673			31			hypothetical protein, unique to CPPV
CPPV135	105670-107640	FGPV088	656	656	hypothetical protein [FGPV]	99.5%	
CPPV136	107582-107977	FGPV089	131	131	putative virion core protein [FGPV]	100.0%	
**CPPV137**	**108258-107974**	**FGPV090**	**130**	**94**	**sulfhydryl oxidase ERV1 [PEPV]**	**98.9%**	
**CPPV138**	**108285-111254**	**FGPV091**	**989**	**989**	**DNA polymerase [FGPV]**	**99.7%**	
**CPPV139**	**112094-111246**	**FGPV092**	**282**	**282**	**hypothetical protein [FGPV]**	**99.2%**	
**CPPV140**	**113802-112087**	**FGPV093**	**571**	**571**	**hypothetical protein [FGPV]**	**100.0%**	
CPPV141	116913-113929	FGPV094	1893	994	VARV B22R family protein [FGPV]	99.4%	
CPPV142	120044-117168	FGPV095	1802	958	VARV B22R family protein [FGPV]	94.6%	
**CPPV143**	**120050-120142**			**30**	**poly(A) polymerase large subunit PAPL [ALPV]**	**86.7%**	
CPPV144	125521-120107	FGPV096	1935	1804	VARV B22R family protein [FGPV]	97.9%	
CPPV145	125511-125606			31			hypothetical protein, unique to CPPV, containing a transmembrane helix
CPPV146	131675-125769	FGPV096	1935	1968	VARV B22R family protein [FGPV]	95.8%	
**CPPV147**	**131742-132290**	**FGPV097**	**182**	**182**	**RNA polymerase subunit 30 [FGPV]**	**100.0%**	
**CPPV148**	**132345-134498**	**FGPV098**	**717**	**717**	**hypothetical protein [FGPV]**	**99.0%**	

CPPV Synteny	CPPV Genome Coordinates	FGPV Synteny	FGPV AA size	CPPV AA size	Best BLAST hits	CPPV AA Identity (%) compared to avipoxviruses	notes
**CPPV149**	**134485-135903**	**FGPV099**	**472**	**472**	**polyA polymerase large subunit PAP-L [FeP2]**	**99.6%**	
CPPV150	136241-135897	FGPV100	114	114	DNA-binding protein [FWPV]	100.0%	
CPPV151	136318-136950	FGPV101	210	210	hypothetical protein [FGPV]	98.6%	
CPPV152	137071-137520	FGPV102	144	149	hypothetical protein [PEPV]	100.0%	
CPPV153	137565-137470			31			hypothetical protein, unique to CPPV, containing a transmembrane helix
CPPV154	137669-137761			30			hypothetical protein, unique to CPPV
CPPV155	137748-138047	FGPV103	99	99	hypothetical protein [FGPV]	97.0%	
CPPV156	143439-138103	FGPV104	1779	1778	VARV B22R family protein [FGPV]	98.2%	
**CPPV157**	**143622-144755**	**FGPV105**	**377**	**377**	**virion envelope protein [FeP2]**	**98.4%**	
**CPPV158**	**144793-146712**	**FGPV106**	**639**	**639**	**virion release protein [FGPV]**	**98.4%**	
CPPV159	146758-148116	FGPV107	452	452	hypothetical protein [FGPV]	97.8%	
**CPPV160**	**148195-149529**	**FGPV108**	**444**	**444**	**serine/threonine protein kinase [FGPV]**	**99.3%**	
**CPPV161**	**149504-150145**	**FGPV109**	**213**	**213**	**lipid membrane protein [FGPV]**	**98.6%**	
CPPV162	150235-150435	FGPV110	66	66	hypothetical protein [PEPV]	100.0%	
CPPV163	150653-150549			34	virion core protein [ALPV]	73.5%	
CPPV164	150633-150833			66	hypothetical protein [FWPV]	92.9%	
CPPV165	150826-151377	FGPV111	174	183	HAL3 domain protein [PEPV]	99.5%	
CPPV166	151451-153760	FGPV112	769	769	ankyrin repeat protein [FGPV]	99.4%	
CPPV167	153800-155422			540	ankyrin repeat protein [PEPV]	93.9%	

**CPPV Synteny**	**CPPV Genome Coordinates**	**FGPV Synteny**	**FGPV AA size**	**CPPV AA size**	**Best BLAST hits**	**CPPV AA Identity (%) compared to avipoxviruses**	**notes**
CPPV168	155448-155816	FGPV113	123	122	CC-chemokine family protein [FGPV]	98.4%	
CPPV169	155931-155833			32			hypothetical protein, unique to CPPV, containing a transmembrane helix
**CPPV170**	**156358-157680**	**FGPV114**	**440**	**440**	**flap endonuclease [FGPV]**	**99.6%**	
**CPPV171**	**157682-157873**	**FGPV115**	**63**	**63**	**RNA polymerase subunit RPO7 [PEPV]**	**100.0%**	
CPPV172	157873-158439	FGPV116	188	188	hypothetical protein [FGPV]	100.0%	
**CPPV173**	**159435-158404**	**FGPV117**	**343**	**343**	**virion core protein [FGPV]**	**99.7%**	
**CPPV174**	**159663-160265**	**FGPV118**	**174**	**200**	**hypothetical protein [FGPV]**	**87.6%**	
**CPPV175**	**160725-161756**	**FGPV119**	**341**	**343**	**N1R/p28-like protein [ALPV]**	**77.7%**	
CPPV176	161996-163021			341	N1R/p28-like protein [SWPV2]	87.3%	
CPPV177	163058-162963			31			hypothetical protein, unique to CPPV
CPPV178	163429-163836			135	hypothetical protein [TKPV]	41.0%	
CPPV179	163899-164000			33			hypothetical protein, unique to CPPV
CPPV180	164131-164021			36			hypothetical protein, unique to CPPV
**CPPV181**	**164179-165246**			**355**	**N1R/p28-like protein [FWPV]**	**75.9%**	
CPPV182	167475-166429			348	TGF-beta-like protein [CNPV]	92.8%	
CPPV183	167524-167976			150	TGF-beta-like protein [CNPV]	74.7%	
CPPV184	168539-169576	FGPV120	357	345	V-type Ig domain protein [FGPV]	99.1%	

**CPPV Synteny**	**CPPV Genome Coordinates**	**FGPV Synteny**	**FGPV AA size**	**CPPV AA size**	**Best BLAST hits**	**CPPV AA Identity (%) compared to avipoxviruses**	**notes**
CPPV185	169848-170333			161	Ig-like domain protein [CNPV]	88.3%	
CPPV186	170433-171488			351	N1R/p28-like protein [CNPV]	95.0%	
CPPV187	171561-172211	FGPV122	217	216	thymidylate kinase [FGPV]	96.3%	
**CPPV188**	**172250-173032**	**FGPV123**	**260**	**260**	**late transcription factor VLTF-1 [PEPV]]**	**99.6%**	
**CPPV189**	**173045-174055**	**FGPV124**	**336**	**336**	**myristylated protein [PEPV]**	**99.4%**	
**CPPV190**	**174056-174787**	**FGPV125**	**243**	**243**	**myristylated MV membrane protein [FGPV]**	**100.0%**	
**CPPV191**	**174822-175112**	**FGPV126**	**95**	**96**	**hypothetical protein [PEPV]**	**96.9%**	
**CPPV192**	**176007-175102**	**FGPV127**	**297**	**301**	**hypothetical protein [PEPV]**	**99.0%**	
**CPPV193**	**176033-176794**	**FGPV128**	**253**	**253**	**DNA binding virion core VP8 [FGPV]**	**99.2%**	
CPPV194	176795-177184	FGPV129	129	129	hypothetical protein [FGPV]	100.0%	
**CPPV195**	**177135-177581**	**FGPV130**	**148**	**148**	**hypothetical protein [FGPV]**	**100.0%**	
**CPPV196**	**177614-178540**	**FGPV131**	**308**	**308**	**polyA polymerase small subunit [FGPV]**	**99.4%**	
**CPPV197**	**178537-179097**	**FGPV132**	**186**	**186**	**RNA polymerase subunit 22 [FGPV]**	**100.0%**	
**CPPV198**	**179500-179087**	**FGPV133**	**137**	**137**	**membrane protein [FGPV]**	**100.0%**	
**CPPV199**	**179541-183404**	**FGPV134**	**1287**	**1287**	**RNA polymerase subunit 147 [FGPV]**	**99.9%**	
**CPPV200**	**183910-183410**	**FGPV135**	**166**	**166**	**protein tyrosine phosphatase [FeP2]**	**100.0%**	
**CPPV201**	**183926-184498**	**FGPV136**	**190**	**190**	**hypothetical protein [PEPV]**	**100.0%**	
**CPPV202**	**185656-184655**	**FGPV137**	**333**	**333**	**virion envelope protein p35 [FGPV]**	**99.4%**	
**CPPV203**	**188056-185657**	**FGPV138**	**799**	**799**	**RNA polymerase-associated protein 94 [FGPV]**	**99.5%**	
**CPPV204**	**188202-188720**	**FGPV139**	**172**	**172**	**late transcription factor 4 [FGPV]**	**98.8%**	
**CPPV205**	**188721-189671**	**FGPV140**	**318**	**316**	**DNA topoisomerase [PEPV]**	**99.4%**	
**CPPV206**	**189676-190134**	**FGPV141**	**152**	**152**	**putative 17 kDa protein [FGPV]**	**100.0%**	
CPPV207	190408-190097	FGPV142	103	103	hypothetical protein [FGPV]	97.6%	

CPPV Synteny	CPPV Genome Coordinates	FGPV Synteny	FGPV AA size	CPPV AA size	Best BLAST hits	CPPV AA Identity (%) compared to avipoxviruses	notes
**CPPV208**	**190416-192974**	**FGPV143**	**852**	**852**	**mRNA capping enzyme large subunit [FGPV]**	**99.7%**	
CPPV209	193055-193369	FGPV144	109	104	HT motif family protein [FGPV]	100.0%	
**CPPV210**	**193788-193366**	**FGPV145**	**140**	**140**	**virion protein [FGPV]**	**100.0%**	
CPPV211	193854-193946			30			hypothetical protein, unique to CPPV
CPPV212	194117-194686			189	hypothetical protein [PEPV]	96.3%	
CPPV213	194739-195593	FGPV146	284	284	N1R/p28-like protein [FGPV]	98.2%	
CPPV214	195635-196357	FGPV147	238	240	deoxycytidine kinase [FGPV]	99.6%	
CPPV215	196746-196363	FGPV148	127	127	HT motif family protein [FGPV]	100.0%	
CPPV216	196842-197471	FGPV149	208	209	hypothetical protein [FGPV]	99.0%	
CPPV217	197512-198339	FGPV150	275	275	N1R/p28-like protein [FGPV]	98.9%	
CPPV218	198368-199600	FGPV151	410	410	N1R/p28-like protein [FGPV]	97.6%	
CPPV219	199649-199810			53	hypothetical protein [FWPV]	84.9%	
CPPV220	199832-200242	FGPV152	136	136	HT motif family protein [FGPV]	97.1%	
CPPV221	200288-201274	FGPV153	328	328	N1R/p28-like protein [FGPV]	98.5%	
CPPV222	201329-202723	FGPV154	464	464	photolyase [FGPV]	99.6%	
CPPV223	202668-202808			46			hypothetical protein, unique to CPPV
CPPV224	202861-203601	FGPV155	246	246	N1R/p28-like protein [FGPV]	99.2%	
CPPV225	203682-204152	FGPV156	156	156	hypothetical protein [FGPV]	99.4%	
CPPV226	204204-204653	FGPV157	149	149	N1R/p28-like protein [FGPV]	100.0%	
CPPV227	204691-205107	FGPV158	133	138	N1R/p28-like protein [FGPV]	98.5%	
CPPV228	205141-205278			45	hypothetical protein [PEPV]	100.0%	

CPPV Synteny	CPPV Genome Coordinates	FGPV Synteny	FGPV AA size	CPPV AA size	Best BLAST hits	CPPV AA Identity (%) compared to avipoxviruses	notes
CPPV229	205294-205776	FGPV159	160	160	N1R/p28-like protein [FeP2]	97.5%	
CPPV230	205806-205922			38			hypothetical protein, unique to CPPV
CPPV231	206292-206435			47	ankyrin repeat protein [FGPV]	59.0%	
CPPV232	206458-206616			52	ankyrin repeat protein [SWPV1]	48.9%	
CPPV233	206716-208518	FGPV160	611	600	ankyrin repeat protein [PEPV]	94.5%	
CPPV234	208638-209408	FGPV161	249	256	N1R/p28-like protein [FGPV]	96.9%	
CPPV235	209456-211663	FGPV162	741	735	ankyrin repeat protein [FGPV]	99.3%	
CPPV236	211756-212907	FGPV163	379	383	hypothetical protein [FGPV]	96.1%	
CPPV237	212937-213908	FGPV164	302	323	ribonucleotide reductase small subunit [FGPV]	99.3%	
CPPV238	213930-214022			30			hypothetical protein, unique to CPPV
CPPV239	214086-215093			335	ankyrin repeat containing protein [FIPV]	79.2%	
CPPV240	215199-215414			71	ankyrin repeat protein [ChePV1]	84.5%	
CPPV241	215431-215309			40	ankyrin repeat protein [PEPV2]	73.5%	
CPPV242	**216101-215424**	**FGPV165**	**225**	**225**	**late transcription factor 3 [FGPV]**	**99.1%**	
**CPPV243**	**216319-216098**	**FGPV166**	**72**	**73**	**virus redox protein [PEPV]**	**98.6%**	
**CPPV244**	**218314-216335**	**FGPV167**	**660**	**659**	**virion core protein P4b [FGPV]**	**99.7%**	
**CPPV245**	**219222-218398**	**FGPV168**	**265**	**274**	**immunodominant virion protein [FGPV]**	**97.7%**	
**CPPV246**	**219261-219770**	**FGPV169**	**169**	**169**	**RNA polymerase subunit RPO19 [PEPV]**	**100.0%**	
**CPPV247**	**220889-219765**	**FGPV170**	**374**	**374**	**hypothetical protein [FGPV]**	**98.9%**	
**CPPV248**	**223025-220896**	**FGPV171**	**709**	**709**	**early transcription factor large subunit [FGPV]**	**98.9%**	

**CPPV Synteny**	**CPPV Genome Coordinates**	**FGPV Synteny**	**FGPV AA size**	**CPPV AA size**	**Best BLAST hits**	**CPPV AA Identity (%) compared to avipoxviruses**	**notes**
**CPPV249**	**223091-223996**	**FGPV172**	**301**	**301**	**intermediate transcription factor VITF-3 [PEPV]**	**99.7%**	
**CPPV250**	**224188-223958**	**FGPV173**	**76**	**76**	**hypothetical protein [FWPV]**	**100.0%**	
**CPPV251**	**226861-224189**	**FGPV174**	**891**	**890**	**virion core protein P4a [PEPV]**	**99.1%**	
**CPPV252**	**226882-227706**	**FGPV175**	**273**	**274**	**hypothetical protein [PEPV]**	**99.3%**	
**CPPV253**	**228231-227707**	**FGPV176**	**175**	**174**	**virion protein [PEPV]**	**98.8%**	
**CPPV254**	228246-228458	FGPV177	70	70	hypothetical protein [PEPV]	95.7%	
CPPV255	**228657-228442**	**FGPV178**	**71**	**71**	**virion membrane protein [FGPV]**	**98.6%**	
**CPPV256**	**228999-228724**	**FGPV179**	**91**	**91**	**virion envelope protein [PEPV]**	**98.9%**	
CPPV257	229177-229016	FGPV180	53	53	A14.5L IMV membrane virulence protein [FWPV]	100.0%	
**CPPV258**	**229486-229193**	**FGPV181**	**97**	**97**	**hypothetical protein [FeP2]**	**97.9%**	
**CPPV259**	**230579-229470**	**FGPV182**	**368**	**369**	**putative mystirilated membrane protein [PEPV]**	**95.7%**	
**CPPV260**	**231191-230595**	**FGPV183**	**198**	**198**	**phosphorylated virion membrane protein [FGPV]**	**100.0%**	
**CPPV261**	**231209-232597**	**FGPV184**	**462**	**462**	**DNA helicase [FGPV]**	**98.5%**	
**CPPV262**	**232831-232565**	**FGPV185**	**88**	**88**	**hypothetical protein [PEPV]**	**97.7%**	
**CPPV263**	**233180-232839**	**FGPV186**	**113**	**113**	**hypothetical protein [FGPV]**	**98.2%**	
**CPPV264**	**233179-234477**	**FGPV187**	**432**	**432**	**processivity factor [PEPV]**	**98.8%**	
**CPPV265**	**234477-234947**	**FGPV188**	**161**	**156**	**hypothetical protein [PEPV]**	**99.4%**	
**CPPV266**	**234958-236109**	**FGPV189**	**383**	**383**	**intermediate transcription factor 3 [FGPV]**	**98.2%**	
**CPPV267**	**236136-239609**	**FGPV190**	**1157**	**1157**	**RNA polymerase subunit RPO132 [FeP2]**	**99.6%**	
CPPV268	241439-239598	FGPV191	612	613	A-type inclusion protein [FGPV]	98.7%	
CPPV269	242899-241475	FGPV192	474	474	A-type inclusion protein [FGPV]	99.6%	

**CPPV Synteny**	**CPPV Genome Coordinates**	**FGPV Synteny**	**FGPV AA size**	**CPPV AA size**	**Best BLAST hits**	**CPPV AA Identity (%) compared to avipoxviruses**	**notes**
**CPPV270**	**243322-242900**	**FGPV193**	**140**	**140**	**hypothetical protein [FGPV]**	**100.0%**	
**CPPV271**	**244245-243337**	**FGPV194**	**302**	**302**	**RNA polymerase subunit 35 [FGPV]**	**98.7%**	
**CPPV272**	**244444-244220**	**FGPV195**	**74**	**74**	**hypothetical protein [FeP2]**	**100.0%**	
CPPV273	244612-244499	FGPV196	37	37	A30.5L-like protein [PEPV]	100.0%	
CPPV274	244622-244963	FGPV197	113	113	hypothetical protein [PEPV]	99.1%	
CPPV275	244964-245326	FGPV198	120	120	hypothetical protein [FeP2]	97.5%	
**CPPV276**	**246229-245315**	**FGPV199**	**304**	**304**	**virion assembly protein [FGPV]**	**98.7%**	
**CPPV277**	**246406-246927**	**FGPV200**	**173**	**173**	**C-type lectin-like protein [PEPV]**	**97.1%**	
CPPV278	246987-247649	FGPV201	220	220	V-type Ig domain protein [FGPV]	96.8%	
CPPV279	247624-248418	FGPV202	263	264	V-type Ig domain protein [FGPV]	97.0%	
CPPV280	248457-249290	FGPV203	277	277	hypothetical protein [FGPV]	97.8%	
CPPV281	249369-250202	FGPV204	285	277	tyrosine protein kinase [FGPV]	96.8%	
CPPV282	250241-251269	FGPV205	342	342	serpin family protein [FGPV]	98.5%	
CPPV283	251942-251280	FGPV206	220	220	hypothetical protein [FGPV]	98.6%	
CPPV284	252049-252975	FGPV207	308	308	G-protein-coupled receptor family protein [FGPV]	99.4%	
CPPV285	252987-253289	FGPV208	92	100	hypothetical protein [FWPV]	92.0%	
CPPV286	253348-253887	FGPV209	179	179	beta-NGF-like family protein [FGPV]	97.2%	
CPPV287	254301-253888	FGPV210	137	137	HT motif family protein [FGPV]	94.2%	
CPPV288	254351-254989	FGPV211	212	212	hypothetical protein [FGPV]	99.5%	
CPPV289	255425-255000	FGPV212	145	141	HT motif family protein [FGPV]	96.6%	
CPPV290	255501-255827	FGPV213	108	108	CC-chemokine family protein [FGPV]	99.1%	
CPPV291	255882-256355	FGPV214	157	157	putative interleukin binding protein [TePV1]	31.0%	
CPPV292	256345-256818	FGPV215	303	157	epidermal growth factor-like protein [FGPV]	96.2%	

CPPV Synteny	CPPV Genome Coordinates	FGPV Synteny	FGPV AA size	CPPV AA size	Best BLAST hits	CPPV AA Identity (%) compared to avipoxviruses	notes
CPPV293	256821-257732	FGPV216	162	303	serine/threonine protein kinase [FeP2]	96.0%	
CPPV294	257786-258274			162	hypothetical protein [FGPV]	97.5%	
CPPV295	258280-258723	FGPV217	124	147	putative 13.7 kDa protein [FGPV]	99.2%	
CPPV296	258767-258862			31			hypothetical protein, unique to CPPV
CPPV297	258832-259056	FGPV218	74	74	hypothetical protein [PEPV]	100.0%	
CPPV298	259123-259287			54			hypothetical protein, unique to CPPV
CPPV299	259297-259818			173	hypothetical protein [FWPV]	79.5%	
CPPV300	259969-260853	FGPV219	294	294	ankyrin repeat protein [FGPV]	99.7%	
CPPV301	260903-261334	FGPV220	143	143	host range protein [FGPV]	99.3%	
CPPV302	261375-262358			327	hypothetical protein [FWPV]	89.0%	
CPPV303	262396-262611			71	ankyrin repeat protein [FGPV]	100.0%	
CPPV304	262992-263780	FGPV221	475	262	ankyrin repeat protein [FGPV]	97.7%	
CPPV305	263786-265114	FGPV222	442	442	ankyrin repeat protein [FGPV]	98.6%	
CPPV306	265667-265116	FGPV223	97	183	hypothetical protein [FGPV]	100.0%	
CPPV307	265761-267995	FGPV224	183	744	ankyrin repeat protein [FeP2]	89.8%	
CPPV308	268051-269727	FGPV225	745	558	ankyrin repeat containing protein [FIPV]	48.7%	
CPPV309	269768-270658	FGPV226	292	296	serine/threonine protein kinase [PEPV]	96.3%	
CPPV310	270726-271811	FGPV227	361	361	ankyrin repeat protein [FGPV]	99.5%	
CPPV311	271913-273460	FGPV228	502	515	ankyrin repeat protein [FWPV]	84.7%	
CPPV312	273495-274451			318	ankyrin repeat protein [TePV1]	40.5%	
CPPV313	274500-276008			502	ankyrin repeat protein [FGPV]	99.4%	

CPPV Synteny	CPPV Genome Coordinates	FGPV Synteny	FGPV AA size	CPPV AA size	Best BLAST hits	CPPV AA Identity (%) compared to avipoxviruses	notes
CPPV314	276057-277103	FGPV229	348	348	serpin family protein [FGPV]	99.1%	
CPPV315	277657-277100	FGPV230	185	185	putative A47L-like protein [FGPV]	99.5%	
CPPV316	277787-279298	FGPV231	503	503	ankyrin repeat protein [FGPV]	99.2%	
CPPV317	279348-280793	FGPV232	489	481	ankyrin repeat protein [FGPV]	98.8%	
CPPV318	280837-282345	FGPV233	502	502	ankyrin repeat protein [FGPV]	99.8%	
CPPV319	282399-283691	FGPV234	430	430	ankyrin repeat protein [FGPV]	99.1%	
CPPV320	283709-284155	FGPV235	148	148	C-type lectin family protein [FGPV]	98.7%	
CPPV321	284158-284985	FGPV236	275	275	N1R/p28-like protein [FGPV]	98.2%	
CPPV322	285090-285308	FGPV237	72	72	putative P-type ATPase [FGPV]	76.4%	
CPPV323	285803-285312	FGPV238	163	163	C-type lectin family protein [FGPV]	98.8%	
CPPV324	285921-287156	FGPV240	411	411	ankyrin repeat protein [FGPV]	98.8%	
CPPV325	287179-287409			76			hypothetical protein, unique to CPPV
CPPV326	287464-287592			42			hypothetical protein, unique to CPPV
CPPV327	287785-288261	FGPV239	88	158	conserved hypothetical protein [SWPV1]	36.8%	
CPPV328	288291-288923	FGPV242	210	210	hypothetical protein [FGPV]	100.0%	
CPPV329	289122-290906	FGPV243	643	594	ankyrin repeat protein [FGPV]	99.3%	
CPPV330	290896-291474	FGPV244	192	192	ankyrin repeat protein [FGPV]	99.5%	
CPPV331	291530-292921	FGPV245	463	463	V-type Ig domain protein [FGPV]	97.4%	
CPPV332	292956-294956	FGPV246	667	666	ankyrin repeat protein [FGPV]	93.7%	
CPPV333	296287-294953	FGPV247	444	444	ankyrin repeat protein [FGPV]	98.7%	
CPPV334	296427-297194			255	immunoglobulin-like domain protein [FIPV]	63.0%	

CPPV Synteny	CPPV Genome Coordinates	FGPV Synteny	FGPV AA size	CPPV AA size	Best BLAST hits	CPPV AA Identity (%) compared to avipoxviruses	notes
CPPV335	297233-297940			235	Immunoglobulin-like domain protein [CNPV]	59.4%	
CPPV336	298032-299786	FGPV248	584	584	ankyrin repeat protein [FGPV]	96.9%	
CPPV337	299786-299887			33			hypothetical protein, unique to CPPV
CPPV338	299866-300240	FGPV249	124	124	EFc family protein [FGPV]	99.2%	
CPPV339	300359-300808	FGPV250	149	149	N1R/p28-like protein [FGPV]	99.3%	
CPPV340	300877-302685	FGPV251	110	602	ankyrin repeat protein [FWPV]	36.1%	
CPPV341	302773-302868			31			hypothetical protein, unique to CPPV
CPPV342	303133-302954			59	ankyrin repeat protein [PEPV]	91.5%	
CPPV343	303328-303218			36	V-type Ig domain protein [FGPV]	56.8%	
CPPV344	303440-303817			125	Ig-like domain protein [SWPV1]	65.3%	
CPPV345	304222-305547			441	ankyrin repeat protein [FWPV]	95.7%	
CPPV346	305634-305524			36			hypothetical protein, unique to CPPV, containing a transmembrane helix
CPPV347	307137-305764			457	ankyrin repeat protein [FWPV]	95.8%	
CPPV348	307759-307244	FGPV252	124	171	C-type lectin protein [FWPV]	92.3%	
CPPV349	308316-310157	FGPV253	508	613	ankyrin repeat protein [SWPV1]	73.6%	
CPPV350	310335-310544			69	immunoglobulin-like domain protein [FWPV]	75.4%	
CPPV351	310562-310660			32	C-type lectin family protein [FGPV]	93.8%	

CPPV Synteny	CPPV Genome Coordinates	FGPV Synteny	FGPV AA size	CPPV AA size	Best BLAST hits	CPPV AA Identity (%) compared to avipoxviruses	notes
CPPV352	311334-310690	FGPV254	201	214	conserved hypothetical protein [SWPV2]	93.2%	
CPPV353	311821-311153			222	hypothetical protein [FIPV]	97.3%	
CPPV354	312118-312008			36			hypothetical protein, unique to CPPV, containing a transmembrane helix
CPPV355	312341-312496			51	C-type lectin-like protein [CNPV]	88.2%	
CPPV356	313242-312766			158	hypothetical protein [FIPV]	96.2%	
CPPV357	313665-313447			72	hypothetical protein [FIPV]	83.9%	

Note: CPPV, cooks petrelpox virus; ALPV, albatrosspox virus; FGPV, flamingopox virus; PEPV, penguinpox virus; FeP2, pigeonpox virus; CNPV, canarypox virus; FIPV, finch poxvirus; FWPV, fowlpox virus; TePV1, teiidae poxvirus 1; ChePV1, cheloniidpox virus 1; SWPV2, shearwaterpox virus 2; SWPV1, shearwaterpox virus 1.

Bold: The 87 core genes conserved in all ChPVs which are involved in essential functions such as replication, transcription and virion assembly.

Green shaded: An additional 47 ORFs found to be uniquely conserved in the selected fully sequenced avian poxvirus genomes.

Supplementary Table S2. 47 ORFs found to be uniquely conserved in the selected fully sequenced avian poxvirus genomes

**CPPR**	**CNPV**	**FeP2**	**PEPV**	**CRPV**	**ALPV2**	**FWPV**	**MPPV2**	**MPPV**	**SWPV2**	**SWPV1**	**TKPV**	**Function**
36	32	19	19	48	33	16	44	34	28	24	001.1a	Ig-like domain
37	33	20	20	49	34	17	45	35	29	25	2	V-type Ig domain
42	38	24	24	57	40	20	53	41	34	28	5	C4L/C10L protein
43	39	25	25	58	41	21	54	42	35	29	6	GPCR
44	40	26	26	59	42	22	55	43	36	30	7	Ankyrin repeat
46	41	27	27	61	43	23	57	44	37	31	8	Ankyrin repeat
47	42	28	28	62	44	24	58	45	38	32	9	Ankyrin repeat
57	48	35	35	69	53	30	66	52	44	38	12	Alkaline phosphodiesterase
58	50	36	36	71	54	31	69	55	46	40	13	Ankyrin repeat
63	53	38	40	74	60	35	72	58	49	44	16	Hypothetical protein
65	55	39	41	77	62	37	74	60	51	46	17	Hypothetical protein
67	58	41	43	80	64	39	77	63	54	49	20	B-cell lymphoma 2 (Bcl-2)
68	59	42	44	81	65	40	78	64	55	50	21	Serpin
70	61	44	46	83	69	43	81	66	57	52	22	DNA ligase
71	62	45	47	84	70	44	82	67	58	53	23	Serpin family
72	63	46	48	85	71	46	83	68	59	54	24	Hydroxysteroid dehydrogenase
74	65	47	49	88	73	47	87	71	61	56	25	Semaphorin
77	68	48	50	91	76	48	92	75	64	59	26	GNS1/SUR4
83	76	54	56	98	82	54	103	83	72	66	32	mutT motif
103	88	65	67	120	96	65	-	-	83	78	40	Hypothetical protein
106	92	68	70	125	100	68	128	98	87	82	42	Hypothetical protein
108	94	70	72	128	102	70	130	100	89	84	44	T10-like protein
111	97	72	75	131	104	71	-	104	92	87	46	Hypothetical protein
117	103	77	78	137	109	75	140	110	98	92	50	N1R/p28

CPPR	CNPV	FeP2	PEPV	CRPV	ALPV2	FWPV	MPPV2	MPPV	SWPV2	SWPV1	TKPV	Function
128	113	87	89	148	120	86	150	120	108	102	60	Thymidine kinase
135	118	93	95	154	126	91	156	126	113	107	65	Hypothetical protein
136	119	94	96	155	127	92	157	127	114	108	66	virion core protein
151	131	106	108	167	142	104	169	139	126	120	75	Hypothetical protein
152	132	107	109	168	143	105	170	140	127	121	76	Hypothetical protein
159	137	112	114	173	149	110	175	145	132	126	80	Hypothetical protein
162	140	115	117	176	152	113	178	148	135	129	83	Hypothetical protein
207	191	146	153	243	196	145	243	199	179	167	109	Hypothetical protein
214	199	153	159	252	203	151	253	209	187	175	113	Deoxycytidine kinase
268	264	195	203	323	255	190	331	274	250	237	140	A-type inclusion protein
269	265	196	204	324	256	191	333	275	251	238	141	A-type inclusion protein
275	270	202	210	329	262	196	339	280	256	243	144	Hypothetical protein
280	273	207	215	333	267	201	343	284	259	247	149	Hypothetical protein
281	274	208	216	334	269	203	344	285	260	248	150	Tyrosine kinase
283	276	210	218	336	271	205	346	287	262	250	151	Hypothetical protein
285	278	212	220	338	273	207	348	289	264	252	151.1a	Hypothetical protein
288	281	214	222	341	277	208	351	292	267	255	152	Hypothetical protein
292	285	216	225	345	280	211	355	296	271	259	153	Epidermal Growth Factor
293	286	217	226	346	281	212	356	297	272	260	154	Serine/threonine protein kinase
294	287	218	227	347	282	213	357	298	273	261	155	Hypothetical protein
295	289	219	228	350	284	214	361	300	275	263	156	Putative 13.7 kDa protein
324	296	226	234	357	293	219	370	308	282	272	161	Ankyrin repeat
349	304	238	248	374	312	232	394	327	290	283	164	Ankyrin repeat

Note: the numbers in each column refer to the specific ORF in each respective genome.

Supplementary Table S3. Number of ORFs in each of the 14 multigene families identified in the fully sequenced avian poxvirus genomes

**Gene family**	**CPPR**	**CRPV**	**ALPV2**	**ALPV**	**FWPV**	**MPPV2**	**MPPV**	**PEPV2**	**CNPV**	**SWPV2**	**MLPV**	**SWPV1**	**FP9**	**PEPV**	**FeP2**	**TKPV**	**FGPV**
Ankyrin Repeat	55		33	48	31	78	62	49	51	46	47	50	22	33	26	16	45
B22R	5	8	6	6	6	9	7	6	6	7	7	6	5	5	4	1	4
N1R/p28	16	23	12	28	10	20	24	24	26	20	25	20	8	11	11	3	13
C4L/C10L	2	5	3	3	3	4	2	3	3	3	3	2	3	2	2	2	2
CC chemokine	8	4	4	5	4	7	4	5	5	5	5	6	4	1	4	2	6
C-type lectin	11	7	8	14	9	11	10	11	11	11	13	13	6	7	4	2	4
G protein-coupled receptor	4	4	3	4	3	4	4	4	4	4	4	4	2	3	2	2	3
HT motif	8	5	6	5	6	5	5	5	5	4	5	4	6	5	4	1	7
Ig-like domain	3	9	6	9	5	13	10	9	9	8	8	9	4	6	4	3	9
Serpin	5	5	6	5	5	5	5	5	5	5	5	5	5	4	4	3	5
EFc	1	2	3	2	3	3	2	2	2	2	1	2	2	1	1	1	1
TGF-β	2	6	1	5	1	5	4	5	5	4	6	3	1	1	1	1	1
β-NGF	3	2	2	2	2	2	2	2	2	2	2	2	2	0	0	2	3
IL-18 BP	1	3	2	3	1	3	3	3	3	3	3	3	1	1	0	2	0
**TOTAL**	**124**	**156**	**95**	**139**	**89**	**169**	**144**	**133**	**127**	**124**	**134**	**129**	**71**	**80**	**67**	**41**	**103**
